# Intrinsic properties of cupric oxide nanoparticles enable effective filtration of arsenic from water

**DOI:** 10.1038/srep11110

**Published:** 2015-06-05

**Authors:** Kyle J. McDonald, Brandon Reynolds, K. J. Reddy

**Affiliations:** 1 Trihydro Corporation, Laramie, Wyoming, USA; 2 Department of Ecosystem Science and Management, University of Wyoming, Laramie, Wyoming, USA

## Abstract

The contamination of arsenic in human drinking water supplies is a serious global health concern. Despite multiple years of research, sustainable arsenic treatment technologies have yet to be developed. This study demonstrates the intrinsic abilities of cupric oxide nanoparticles (CuO-NP) towards arsenic adsorption and the development of a point-of-use filter for field application. X-ray diffraction and X-ray photoelectron spectroscopy experiments were used to examine adsorption, desorption, and readsorption of aqueous arsenite and arsenate by CuO-NP. Field experiments were conducted with a point-of-use filter, coupled with real-time arsenic monitoring, to remove arsenic from domestic groundwater samples. The CuO-NP were regenerated by desorbing arsenate via increasing pH above the zero point of charge. Results suggest an effective oxidation of arsenite to arsenate on the surface of CuO-NP. Naturally occurring arsenic was effectively removed by both as-prepared and regenerated CuO-NP in a field demonstration of the point-of-use filter. A sustainable arsenic mitigation model for contaminated water is proposed.

Arsenic (As) is a toxic metalloid element that can be found ubiquitously throughout the Earth’s crust. It enters groundwater via natural and anthropogenic pathways: smelting, combustion of fossil fuels, and various agricultural practices. However, the predominant source of arsenic contamination of groundwater is through the weathering of natural minerals (geogenic)^1^. Studies have shown that different human health problems are associated with exposure to elevated levels of arsenic including discoloration of skin, diabetes, intestinal maladies, carcinogenesis, and ultimately death^2^,^3^,^4^. The widespread nature and severity of health issues associated with geogenic arsenic contamination of drinking water is a chief global health concern^5^. The World Health Organization (WHO) and United States Environmental Protection Agency (US EPA) recommend 10 μg/L of arsenic as a limit for human drinking water. Several countries in six continents (Africa, Asia, Australia, North America, Europe, and South America) have reported high levels of arsenic concentration (>10 μg/L) in their drinking water supplies^6^,^7^,^8^,^9^,^10^,^11^,^12^,^13^,^14^,^15^,^16^.

A variety of arsenic removal technologies exist and extensive reviews of these techniques have been published^17^,^18^,^19^,^20^,^21^,^22^,^23^. Of these removal technologies, the adsorption process (use of a sorbent to filter arsenic from water) is the most common method for the treatment of arsenic laden waters. The most widely tested conventional sorbents include aluminum, iron, manganese, titanium, and zirconium^24^,^25^,^26^,^27^. However, most of the conventional arsenic sorbents are not sustainable due to multiple limitations. These limitations include: insufficient removal of arsenic from water, complexity of pre and post treatment steps, cost of the technology, indiscriminate disposal of used sorbents, growth of pathogens in treated water, and lack of instantaneous measurement of arsenic in treated water^27^,^28^. Thus, development of effective and sustainable sorbents for filtration of toxic arsenic species from drinking water is a research priority.

Nanoparticles of metal oxides have been attracting much of the attention in various applications, including water treatment, due to their unique properties. Nanoparticles have the capability to rapidly remove contaminants within minutes from water with a relatively small dose, which makes their application effective and economical. Studies have shown that copper oxide nanoparticles (CuO-NP) can remove aqueous arsenic species under a wide range of geochemical conditions^12^,^14^,^27^,^29^,^30^,^31^,^32^,^33^. However, most of the arsenic removal processes using nanoparticles, including CuO, were conducted in the laboratory and field confirmation of these nanoparticles is urgently required^26^. In a recent review of CuO nanostructures, Zhang *et al.*^27^ reported potential use of CuO-NP as building blocks, due to their distinctive properties, for future micro/nanoscale devices for various applications; including to filter arsenic from water. Thus, the unique arsenic adsorption and desorption properties of CuO-NP could help develop an effective point-of-use water filter for field applications.

The zero point of charge (ZPC) of CuO-NP, at approximately pH 9.4 ± 0.4, allows for adsorption of arsenic under most naturally occurring drinking water systems^12^. This ZPC also provides the opportunity to regenerate and reuse the CuO-NP following the adsorption of arsenic species. The regeneration process is accomplished by raising the pH above the ZPC of CuO (pH > 9.4), where the surface charge of CuO becomes negative, resulting in desorption of anionic arsenic species (Fig. 1a). The desorption of arsenic from the surface of the CuO-NP may also be attributed, in part, to a competitive effect created by the presence of high concentrations of OH- ions in the wash solution. Following desorption and removal of the arsenic, the CuO-NP can then be reused to readsorb arsenic from contaminated groundwater. This drastically increases the viability of CuO-NP as a practical treatment technique for removing arsenic from water by lowering costs in material, production, and disposal. With the goal of developing a sustainable arsenic removal method, we conducted experiments to 1) characterize the surface of CuO-NP as-prepared, after reaction with As(III) (arsenite) and As(V) (arsenate), and following regeneration, 2) characterize the precipitate formed from the regeneration process and perform a mass balance of the regeneration process, 3) develop a point-of-use filter for arsenic removal with CuO-NP for field testing, and 4) analyze samples from the point-of-use filter using a proven, cost-effective arsenic field test^34^ as a real-time monitor of arsenic concentrations in water.

## Results

### Physical CuO nanoparticle characterization

Physical properties of the synthesized CuO-NP were analyzed using high resolution transmission electron microscopy (HRTEM) and Brunauer-Emmett-Teller (BET) analysis (See Methods). HRTEM analysis shows that the nanoparticles formed spherical and cylindrical shapes as shown in Supplemental Fig. 1. The BET surface area of the CuO-NP gave a specific surface area of 62 m^2^/g. X-ray diffraction (XRD) patterns of CuO-NP as-prepared and following regeneration after the sorption of either As(III) (arsenite) and As(V) (arsenate) show very similar peak patterns suggesting that the crystalline structures of CuO-NP do not change following the adsorption and desorption of arsenic. The XRD patterns for prepared CuO, regenerated CuO following reaction with As(III), and regenerated CuO following reaction with As(V) are shown in Supplemental Fig. 2.

### XPS Analyses

**Figure 1:**
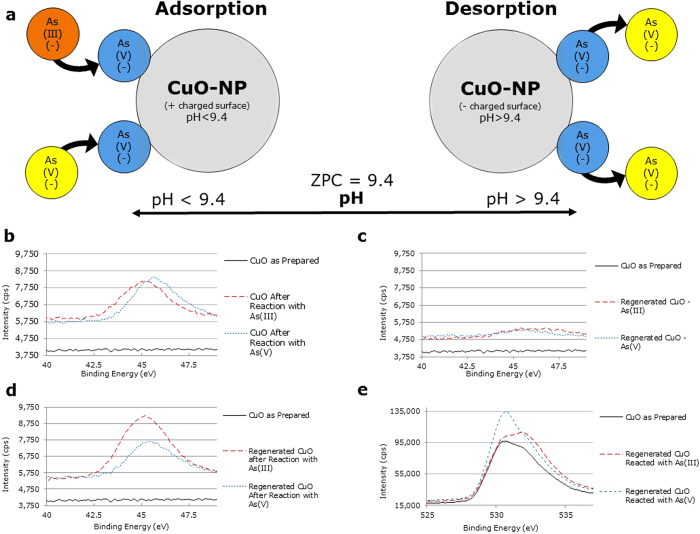
Adsorption schematic and XPS analysis. (**a**) Adsorption and desorption phenomenon of CuO-NP in water. (**b**) XPS spectra at the As3d peak of CuO-NP following initial reaction with As. (**c**) XPS spectra at the As3d peak of CuO-NP following regeneration. (**d**) XPS spectra at the As3d peak of regenerated CuO-NP following reaction with As. (**e**) XPS spectra of the Os1 peak for CuO-NP as-prepared and following regeneration after reaction with either As(III) or As(V).

CuO-NP as-prepared, following sorption of arsenic, following regeneration, and following reuse were analyzed with X-ray photoelectron spectroscopy (XPS). XPS analysis was used to determine the presence or absence of arsenic on the surface of the CuO-NP. This analysis was performed for seven different samples. One sample consisted of CuO-NP as-prepared. The other samples were of CuO-NP following reaction with either As(III) or As(V), CuO-NP following the regeneration process, and regenerated CuO-NP following reaction with either As(III) or As(V). Figure 1b–d compare the As3d XPS spectra at the binding energies ranging from 40 to 49 electron volt (eV). The variation in peaks at this binding energy between the prepared CuO-NP and the CuO-NP following reaction with As(III) and As(V) confirm the adsorption of arsenic onto the surface of the CuO (Fig. 1b). This is similar to results found previously^12^. The decrease in the peaks of the regenerated CuO-NP verifies desorption of arsenic from the CuO-NP surface (Fig. 1c). Further, the reappearance of these peaks for the regenerated CuO-NP following reaction with As(III) and As(V) confirms the adsorption of arsenic onto the surface of the regenerated CuO-NP (Fig. 1d). This data corroborates the ability of CuO-NP to be regenerated and reused after sorption and desorption of aqueous arsenic species.

The As3d binding energies for this data ranged from 45.5 eV to 45.8 eV. No literature for the binding energies of arsenic onto CuO could be found. However, several studies have reported the As3d peak of As(V) occurring in this range of binding energies such as; As(V) on synthetic birnessite located at a binding energy of 45.15 eV^35^, at a binding energy of 45.28 eV for arsenopyrite^36^, as well as As(V) peaks at binding energies of 45.7 through 45.9 eV reported by several other researchers^27^,^37^,^38^,^39^,^40^. For the As3d peak, As(III) binding energies can generally be located at approximately 1 eV lower than the binding energies of As(V) at a range of approximately 44.7 to 45.0 eV^12^,^41^. This is congruent with the results found by Zhang *et al.*^42^ with a Fe-Mn binary oxide adsorbent and two other studies^30^,^32^ with CuO-NP, suggesting that the adsorption process of As(III) involves the oxidation of As(III) to As(V) by the surface of CuO-NP. If CuO is indeed oxidizing As(III) prior to adsorption, we would expect to see the reduction of CuO to Cu_2_O (cuprous oxide). Fig. 1e depicts the O1s peak with XPS spectra plotted for CuO-NP as-prepared, CuO-NP regenerated after reaction with As(III), and CuO-NP regenerated after reaction with As(V). The distinctive peak seen for the CuO-NP reacted with As(V) and the less distinctive humps of CuO-NP as-prepared and CuO-NP reacted with As(III) occur at a binding energy of 530.7 eV. The binding energy for CuO was reported at 530.6 eV^43^ and at 530.7 eV^44^. The less distinctive peak shown in Fig. 1e of the nanoparticles reacted with As(III) occurs at 531.9 eV. Ertl *et al.*^45^ reported the peak of Cu_2_O as being located at a binding energy of 531.1 eV. The presence of multiple peaks, or humps, in the plots shown on Fig. 1e suggests the presence of multiple copper species (Cu^2+^ and Cu^+^). This lends to the proposed electron transfer process near the surface of the CuO-NP resulting in the oxidation of As(III) to As(V) prior to the adsorption by CuO-NP and the subsequent reduction of CuO to Cu_2_O. Yu *et al*^32^ reported similar process of oxidation of As(III) by the electron transfer on the surface of CuO, which could result in the reduction of CuO to Cu_2_O. Thus, the multiple peaks observed in the reaction with As(III) in Fig. 1e were probably due to the presence of CuO and Cu_2_O.

### Arsenic harvesting and mass balance

A mass balance study of arsenic throughout the adsorption and desorption process was conducted using a series of batch experiments. To complete this mass balance analysis of arsenic during the batch experiment process, triplicate water samples were prepared with DI water spiked with equal concentrations of As(III) and As(V) to a pre-treatment concentration of 200 mg/L arsenic. These samples were mixed in with CuO-NP and allowed to react while the pH was maintained at approximately 8. The samples were then decanted and filtered to determine the post-treatment arsenic concentration. The arsenic was then desorbed from the used CuO-NP in the regeneration wash fluids (See Methods). Inductively coupled plasma mass spectrometry (ICP-MS) was used to characterize arsenic concentrations in the pre-treatment water samples, post-treatment water samples, and the regeneration wash fluids. From this data, shown in Supplementary Table 1, an average of 97% of the arsenic was recovered from the batch study regeneration/desorption process.

Regeneration wash fluids containing the desorbed arsenic from the batch study were collected in a weighed crucible and allowed to evaporate. The resulting precipitate was analyzed using X-ray diffraction (XRD). The diffraction patterns for the wash fluid precipitate versus patterns for crystalline sodium arsenate (NaH_2_AsO_4_.H_2_O), sodium arsenite (NaAsO_2_), and sodium hydroxide (NaOH) were compared. Analyses of these patterns suggest that the wash fluid precipitate is largely comprised of sodium hydroxide and sodium arsenate. XRD patterns for sodium hydroxide, sodium arsenate, and sodium arsenite are compared to that of the wash fluid precipitate in Supplementary Fig. 3.

### Point-of-use filter column modifications and set-up

**Figure 2:**
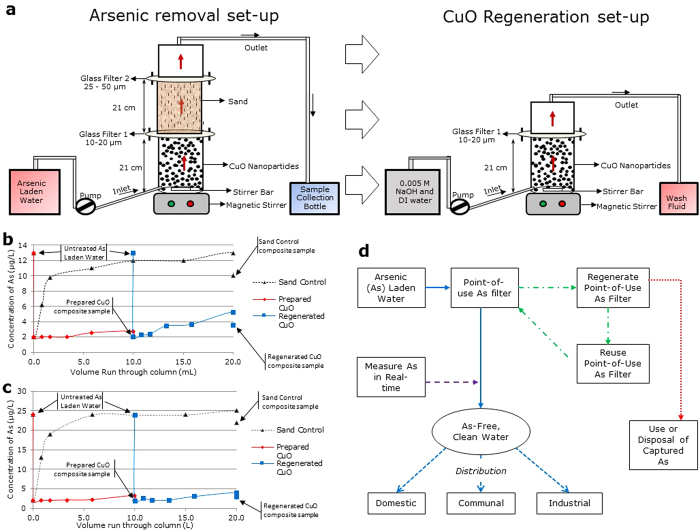
Point-of-use arsenic filter design, field results, and proposed model. **(a)** Point-of use arsenic removal filter set-up and regeneration set-up. **(b)** Removal of arsenic from Torrington domestic groundwater sample with point-of use arsenic removal filter. **(c)** Removal of arsenic from Jackson domestic groundwater sample with point-of use arsenic removal filter. **(d)** A model for sustainable arsenic mitigation process.

Three identical polycarbonate point-of-use filters were designed for lab and field testing (Fig. 2a). The basic design of the filter is derived from previous^14^,^46^ lab experiments. However, several key modifications were made to this column design to allow for it to be operated in the field at ten times the flow rate used in previous lab based studies. (See Methods). Contact time in the point-of-use filter of the CuO-NP and arsenic laden groundwater was approximately 2 min. Pressures were not measured or managed in the flow through reaction column.

Experiments were conducted on three different groundwater samples from Wyoming. For each groundwater sample, three tests were performed; one control (without CuO-NP) and two duplicate runs with CuO-NP. Following the initial treatment of arsenic laden water, the CuO-NP were regenerated in the lab and the field (See Methods). All regeneration wash fluids were collected at the outlet and fully characterized for anion and cation concentrations. After the regeneration process, the flow-through filtration process was repeated using the regenerated CuO-NP. The initial flow-through process using CuO-NP as-prepared, regeneration of the CuO-NP, and second flow through using regenerated CuO-NP were performed. Additionally, control runs with the respective groundwater samples were conducted without CuO-NP in order to determine the effect of the glass filters and sand alone in the point-of-use filter column.

Sampling for all three groundwater samples when treated with CuO-NP was conducted at 0, 5, 10, 20, 35, and 60 minutes, a control sample before testing, and a composite sample of all the water treated by the point-of-use filter were collected during the test. The same sampling protocol was used for the regenerated CuO-NP. The control tests for the three groundwater samples were sampled at 0, 5, 10, 20, 35, 60, 90, 120 min. The difference in sampling protocol was due to the lack of a regeneration step during the control tests. All samples collected were measured for pH, oxidation and reduction potential (ORP), and electrical conductivity (EC) in the lab or field during testing.

### Testing point-of-use filter in the lab with arsenic spiked groundwater

Initial laboratory experiments with the point-of-use filter were conducted using a naturally arsenic-free groundwater sample spiked with As(III) (arsenite) and As(V) (arsenate) to evaluate its performance. This groundwater sample was collected from the Casper Aquifer east of Laramie, Wyoming, USA. The groundwater sample was collected at the wellhead and placed in a reservoir from which it was spiked to approximately 0.1 mg/L arsenic with equal concentrations of As(III) and As(V) (See Methods). The arsenic spiked sample was then pumped from the reservoir through the point-of-user filter column. The chemistry of groundwater spiked with As(III) and As(V) are shown in Supplementary Table 2. The pH of the arsenic spiked groundwater was 7.56. The major element concentrations, in mg/L, were; 72 (Ca), 15 (Mg), 4.1 (Na), 1.0 (K), 11 (Cl), and 19 (SO_4_^2−^). The measured spiked arsenic concentration in the groundwater was 0.094 mg/L. To control the flow rate, the arsenic spiked natural groundwater was placed in a reservoir from which it was pumped through the point-of-use filter column. Water was pumped through the reaction chamber of the point-of-use filter column where the arsenic laden water was reacted with the CuO-NP.

The effect of the glass filters and sand column alone contributes little to the removal of arsenic (Supplementary Table 3). Similar to the results reported by Reddy *et al.*^46^, an initial decrease in arsenic concentrations is seen by the sand column alone. This decrease in arsenic is attributed to the capillary retention of water molecules in the pore spaces of the sand particles. This dilution effect is diminished as more water is pumped through the point-of-use filter column. Results of arsenic concentration and full chemical analysis for the sand only control runs for arsenic spiked groundwater can be found in the Supplementary Table 3. The treatment of CuO-NP as-prepared effectively removed arsenic from the arsenic spiked groundwater sample in the lab. Arsenic concentrations in the spiked groundwater decreased from 0.094 to 0.004 mg/L in the composite sample. An initial drop in pH was observed to 6.70 and rapidly returned to 7.60. Excluding arsenic, no other chemical constituents changed appreciably in the spiked groundwater following the treatment, including copper (Supplementary Table 4). The regenerated CuO-NP were slightly less effective in removing arsenic than the CuO-NP as-prepared. This is due to an incomplete flushing of arsenic from the reaction column in the point-of-use filter during regeneration and subsequently an incomplete regeneration of the CuO-NP. The arsenic concentration of the spiked groundwater decreased from 0.094 to 0.020 mg/L following treatment with the regenerated CuO-NP (Supplementary Table 5).

### Testing point-of-use filter column in the field

For field testing the point-of-use filter was set up on location and the arsenic laden groundwater sample was collected at the wellhead and placed in a reservoir from which it was pumped through the point-of-use filter column. Complete chemistries for the Torrington and Jackson groundwater samples can be found in Supplementary Table 2. The pH of the Torrington and Jackson groundwater samples were 7.22 and 7.50, respectively. The concentration of arsenic at each field location exceeds the 0.01 mg/L maximum contaminant level (MCL) recommended by the USEPA and WHO for drinking water. Arsenic concentrations were 0.013 and 0.024 mg/L in the Torrington groundwater and Jackson groundwater, respectively. The groundwater from Torrington contained concentrations of SO_4_^2−^ of 150 mg/L and Si concentrations of 0.026 mg/L. The groundwater from Jackson contained concentrations of SO_4_^2−^ of 15 mg/L and Si concentrations of 0.008 mg/L.

Figure 2b,c plot the concentration of arsenic versus the volume of water treated using sand alone, CuO-NP as-prepared, and regenerated CuO-NP for the Torrington groundwater and the Jackson groundwater. Similar to the lab point-of-use filter column experiment, the sand and filters alone (control) show no effect on removal of arsenic from Torrington and Jackson groundwater in the field. Results of arsenic concentration and full chemical analysis for the sand only control runs for each location can be found in the Supplementary Table 3. The concentration of arsenic in the Torrington groundwater with as-prepared CuO-NP was decreased from 0.013 to 0.002 mg/L in the composite sample. The pH of the Torrington groundwater prior to treatment was 7.22. A drop in pH was seen to a pH of 6.55 in the initial 0 minute sample following treatment. Following this initial drop the pH slowly increased to a pH of 7.35 in the composite sample. Except for arsenic, no other chemical constituent of the Torrington water changed significantly following the treatment with CuO-NP, including copper. Arsenic concentrations and full chemical analysis for the treatment by CuO-NP as-prepared for Torrington groundwater can be found in the Supplementary Table 4. In the Jackson groundwater, the concentration of arsenic was decreased from 0.024 to 0.002 mg/L. The pH of the Jackson groundwater was 7.50 prior to the treatment with point-of-use filter column. Again, an initial drop in pH to 6.58 was noted, which then increased to 7.40 in the composite sample. There was no significant change in chemical constituents other than arsenic of the Jackson groundwater following the treatment with CuO-NP as-prepared, including copper (Supplementary Table 4). The CuO-NP were regenerated in the field by raising the pH of the solution entering the point-of-use filter column above the ZPC (>pH 9.4 ± 0.4) of CuO-NP, desorbing the arsenic from the CuO-NP into solution which was then flushed through the system (See Methods). The experiments were then repeated with the regenerated CuO-NP. It was noted that the CuO-NP eventually formed aggregates as the water was pumped through the column. The aggregates would break apart during the regeneration process and form again during the regenerated CuO-NP trials. The formation of aggregates could affect adsorption of arsenic and should be studied further.

The regenerated CuO-NP were slightly less effective in removing arsenic, from Torrington and Jackson groundwater samples, than the CuO-NP as-prepared (Figure 2b,c). Similar to observations in the lab based point-of-use filter experiment, this is due to an incomplete flushing of arsenic from the reaction chamber during regeneration and subsequently an incomplete regeneration of the CuO-NP. However, the regeneration process of CuO-NP may be improved by applying a small amount of electrical current. Arsenic concentration in the Torrington groundwater decreased from 0.013 to 0.004 mg/L following the treatment with regenerated CuO-NP, and the Jackson groundwater decreased in arsenic concentration from 0.024 to 0.003 mg/L. The effects of the regenerated CuO-NP on other chemical constituents were similar to that of the CuO-NP as-prepared, with no significant changes observed. A full chemical analysis of the point-of-use filter trials using the regenerated CuO-NP and wash fluids can be found in Supplementary Table 5 and Supplementary Table 6.

Semi quantitative results for twenty select samples were acquired on-site using the Arsenic Low Range Quick field test (Industrial Test Systems, Inc.). The results of the Arsenic Low Range Quick field test were compared to arsenic analysis performed by ICP-MS. The results of the Arsenic Low Range Quick field testing kit are very agreeable with the analytical results retrieved by ICP-MS. Supplementary Fig. 4 plots the results of the semi-quantitative and quantitative analyses against one another. It is clear that the Arsenic Low Range Quick test was very accurate when used in conjunction with the CuO-NP treatment. This suggests that the Arsenic Low Range Quick field test kit could be used as a viable real-time monitor of arsenic concentrations for point-of-use arsenic treatment technologies.

Based on the intrinsic properties of CuO-NP towards aqueous arsenic species, we proposed a model for developing a sustainable arsenic mitigation process using the point-of-use filter (Fig. 2d). As shown in the model, arsenic laden water is passed through the point-of-use filter with CuO-NP to capture arsenic. Since CuO-NP also exhibit anti-bacterial properties, the outlet water will not only be clean from arsenic species but also from pathogens^47^. The outlet water from the point-of-use filter will be monitored for arsenic using an arsenic field testing kit, such as the Arsenic Low Range Quick test, as a real-time measurement. Once the point-of-use filter is saturated with arsenic, it can be regenerated. The regenerated point-of-use filter will be used again to capture arsenic from water. The arsenic collected from regeneration process can then be properly disposed, recycled, or used in industrial process, due to the economic value of arsenic. In addition, the captured arsenic can be easily managed because the volume of regeneration fluids will be minimal. The regeneration process of CuO-NP could also help avoid difficulties in the disposal of used CuO-NP. The arsenic removal point-of-use filter proposed in this study also has the potential to be scaled up to field applications for industrial wastewater containing excess arsenic.

## Discussion

We have demonstrated the mechanism of arsenic adsorption on the surface of CuO-NP and the subsequent desorption via regeneration of the CuO-NP. Batch experiments were conducted to characterize the adsorption of both As(III) and As(V) by prepared CuO-NP and regenerated CuO-NP. XRD analysis shows no change in the crystalline structure of the CuO-NP as-prepared and following regeneration. The initial adsorption, desorption, and second adsorption of arsenic was confirmed by identifying the presence and absence of arsenic on the surface of CuO-NP using XPS. Further, these XPS results suggest that the removal of As(III) by CuO-NP involves the oxidation of As(III) to As(V) prior to adsorption. Several oxidation mechanisms could be involved in this process. However, an electron transfer between the As(III) and CuO-NP is confirmed by XPS analysis of the O1s peak suggesting the reduction of CuO to Cu_2_O. A mass balance of the regeneration process shows a 97% recovery efficiency of arsenic under lab conditions. Additional XRD analysis of the wash fluid precipitate suggests that the composition is largely sodium hydroxide and sodium arsenate.

We have also developed and successfully tested a point-of-use arsenic filter column using CuO-NP in the field that has the ability for on-site regeneration, and demonstrated the application of a cost-effective and accurate real-time arsenic concentration field test kit to be used in conjunction with the point-of-use filter. Experimental results show the successful removal of arsenic from arsenic spiked groundwater in the lab as well as at two locations with naturally high concentrations of arsenic in the field without effect on the other constituents of the water samples including copper. Copper concentrations are monitored closely throughout the treatment process. However, the pH of most naturally occurring water will be sufficiently high that Cu will not dissolve. Though incomplete, the onsite regeneration of the CuO-NP showed a successful recovery of 22% to 38% of the arsenic removed in the initial adsorption process. The regenerated CuO-NP were also successful in removing arsenic from groundwater at all three sites. Overall, the results suggest that CuO-NP, both as-prepared and regenerated, are effective in removing arsenic from water and can perform well under variable and uncontrolled field conditions. The ability for CuO-NP to be regenerated and reused presents an opportunity to significantly lower the operational costs involved in the treatment of arsenic laden water. However, scale up and cost economic studies must be performed. Results found by the use of the Arsenic Low Range Quick field test kit confirm that it is highly accurate and easy to use. Thus it can be readily and efficiently used as a viable real-time monitor of arsenic concentrations in conjunction with the CuO-NP treatment. The conclusion of this research suggest that the unique properties of CuO-NP toward aqueous arsenic species in natural groundwater offer promise in developing a viable and sustainable point-of-use water treatment technology^48^.

## Methods

### Materials

All chemicals used in this study were analytical grade. As(III) and As(V) stock solutions were prepared using Sigma–Aldrich 99% pure NaAsO_2_ and Na_2_HAsO_4_·7H_2_O, respectively, and combined with deionized (DI) water to a concentration of 1000 mg/L each. DI and groundwater samples for laboratory experiments were spiked using these stock solutions to the desired concentrations. NaOH utilized in regeneration was analytical grade. All sample collection bottles were sterile, HDPE plastic.

### CuO nanoparticles

The CuO-NP used in this study were synthesized following the procedures developed by Martinson and Reddy^12^. This method combines solutions of ethanol, copper chloride, sodium hydroxide, and polyethylene glycol in a round bottom flask. The solution is then reacted in a GE model JES1358WL 1200 W microwave that has been modified with a reflux condenser extending from the top at ambient air pressure for 10 minutes at 20% power to generate uniformly sized particles. The supernatant is then cooled to room temperature, decanted, centrifuged and washed with a sequence of DI water at 60–65 °C, ethanol, and acetone. The washed precipitate was then placed in a 110 °C oven to dry and is then ground. This procedure produced spherical and rod shaped CuO-NP that range in size between 12–18 nm in diameter^12^. The synthesized CuO-NP were characterized with High Resolution Transmission Electron Microscopy (HRTEM) and Brunauer-Emmett-Teller (BET) surface area analysis.

### Batch experiment procedure

To perform surface characterization of the CuO-NP following adsorption and to examine the regeneration process, batch experiments were carried out by reacting 50 mL of DI water containing 200 mg/L As(III) and As(V), separately, with 0.5 g CuO-NP.

### Batch experiment regeneration and reuse

To regenerate the CuO-NP in the batch experiment study, first CuO-NP were reacted with As(III) and As(V) in a reaction vessels. Arsenic adsorbed CuO-NP were removed from their reaction vessels and washed with 300 mL of 0.3 M NaOH followed by 300 mL of DI water at 60–65 °C, 100 mL of ethanol, and 100 mL of acetone. The CuO-NP were then placed in a crucible and allowed to dry overnight in a 110 °C oven. During this process, all regeneration wash fluids were collected and stored for analysis.

### Batch experiment wash fluid precipitates

The regeneration wash fluids from the batch experiment were collected and dried to form precipitates. The precipitate was analyzed with XRD. After XRD analysis the precipitate was dissolved with concentrated nitric acid and analyzed with ICP-MS to corroborate XRD results.

### Arsenic spiked groundwater for point-of-use filter lab testing

A groundwater sample containing non-detectable amounts of arsenic was spiked with 50 μg/L As(III) and 50 μg/L As(V) to a concentration of 100 μg/L total arsenic.

### Field point-of-use filter modifications, set up, and sampling

Modifications of the point-of-use filter from previous designs include; construction from polycarbonate, an extended reaction chamber, and an additional flange that isolates the reaction chamber allowing for on-site regeneration of the CuO-NP. Flow rate of the point-of-use filter column was 10 L/hr. The reaction chamber measures 21 cm tall with a 5 cm inner diameter giving the CuO-NP approximately a two-minute contact time with the water. From the reaction chamber the water passes through a glass filter with a 10–20 μm pore size into a column of approximately 300 g of tamped lab grade sand (Fisher Scientific, S25-10, SiO_2_ powder) to filter the CuO-NP. The water then passes through a second glass filter with a pore size of 25–50 μm to the outlet of the column (Fig. 2a).

Setup of the point-of-use filter was atop a portable aluminum table for a work surface. A weighted ring stand was used as assurance to balance the point-of-use filter. The point-of-use filter sat atop a magnetic stir plate that kept the CuO-NP in suspension using a magnetic stir bar inside the reaction chamber. The pump and stir plate were powered using a Duracell 600 W portable battery.

Control tests with sand and no CuO-NP for the point-of-use filter were conducted for 120 min or 20 L water total. Sampling for the point-of-use filter column with CuO-NP was conducted for 60 min or 10 L water both before and after regeneration (for a total of 20 L).

### Field experiment regeneration and reuse

To regenerate the CuO-NP in the field experiments, 1.5 g CuO-NP were reacted with arsenic laden groundwater in the point-of-use filter. After initial filtering, to regenerate the CuO-NP, the sand chamber of the column was removed and 0.005 M NaOH solution was pumped at a flow rate of 10 L/hr through the reaction chamber of the column (Fig. 2a). This molality of NaOH was chosen due to the concern of a highly molar solution precipitating in the column and creating backpressure. In total, 5.68 L of NaOH was pumped through the point-of-use column. This was followed by pumping DI water at a flow rate of 10 L/hr until the pH at the outlet was below 7.50 (between 3.80–5.68 L). After pH below 7.50 was attained, the sand chamber was re-attached to the reaction chamber and filtration from the same groundwater resumed.

### Description of sampling locations

A preliminary flow-through experiment was conducted in the lab using a groundwater sample collected from the Casper aquifer east of Laramie, Wyoming, USA. It was transported to the lab and spiked with 0.05 mg/L As(III) and 0.05 mg/L As(V) to a concentration of 0.1 mg/L total arsenic. Two field locations were chosen for the point-of-use testing of the CuO-NP filter column. These locations were selected due to prior knowledge of the presence of naturally occurring arsenic in concentrations higher than the EPA and WHO drinking water limit of 10 μg/L. The first field site is a groundwater well associated with intensive agricultural activity located near Torrington, Wyoming, USA. The second site is a domestic groundwater well located near Jackson, Wyoming, USA.

### Analytical techniques

The HRTEM images were obtained using a Tecnai G2 F20 200 kV (S)TEM made by FEI Company (Hillsboro, Oregon, USA). Prior to imaging, the samples were placed on a carbon coated copper grid after being dispersed in methanol. Surface area was determined using a TriStar 3000 BET analyzer after being degassed with nitrogen gas for 15 hours. CuO-NP as-prepared and after regeneration were compared using XRD patterns. In addition, the precipitate formed by the regeneration wash fluids was compared to expected possible precipitates using XRD patterns. Powder XRD data were measured at room temperature on a Bruker Apex II diffractometer equipped with a CCD area detector, graphite monochromator and a Mo fine-focus sealed tube (λ = 0.71073 Å) operated at 50 kV and 30 mA. The detector was placed at a distance of 6.13 cm from the sample. A thick paste of the sample was made fromapproximately 10 mg of the sample and paratone N oil, and a ball of approximate size 0.5 × 0.5 × 0.5 mm^3^ was prepared and mounted on a glass fiber. Four to six images of diffraction were measured over a long two-theta range with an exposure time of 3 min per image. The XPS spectra were obtained using a Kratos Axis Ultra DLD X-ray Photoelectron Spectrometer (XPS) with a monochromated Al K-alpha source (1486 eV) running at 150 W. Survey scans were performed using a pass energy of 80 eV and a step size of 1 eV. High resolution scans were performed using a pass energy of 40 eV and a step size of 0.1 eV.

The water samples collected throughout the flow-through column filtration processes were analyzed for major and trace elements. Aliquots of these samples were acidified to a pH of approximately 2 using concentrated trace metal grade nitric acid in the field for cation analysis, while portions were kept at 4 °C and unacidified for anion analysis. ICP-MS was used to determine concentrations of Ca, Na, Mg, K, As, Cr, Si, Cu, Fe, Mn, Pb, and Se. Unacidified samples were analyzed for SO_4_^2−^, Cl^−^, NO_3_^−^, and PO_4_^3−^ concentrations with ion chromatography (IC). More details about model number and detection limits of these two instruments are given in the supplemental information (Supplementary Table 7). An Orion FiveStar multimeter was used to measure pH, EC and OPR on site at the time of collection. In addition, the control, composite, and regeneration wash fluid samples were analyzed on-site using the Arsenic Low Range Quick field testing kit as a real time monitor of arsenic concentrations.

## Additional Information

**How to cite this article**: McDonald, K. J. *et al.* Intrinsic properties of cupric oxide nanoparticles enable effective filtration of arsenic from water. *Sci. Rep.* **5**, 11110; doi: 10.1038/srep11110 (2015).

## Supplementary Material

Supplementary Information
