# Supplementary material for: Intrinsic properties of cupric oxide nanoparticles enable effective filtration of arsenic from water
Source: Sci Rep. 2015 Jun 5;5:11110. doi: 10.1038/srep11110 (PMC4457161; doi:10.1038/srep11110)
Supplement: Supplementary Information [file srep11110-s1.doc]

**Intrinsic properties of cupric oxide nanoparticles enable effective filtration of arsenic from water**

Kyle J. McDonald^1^, Brandon Reynolds^2^, Katta Jayaram Reddy^2,3,*^

1. Trihydro Corporation, Laramie, Wyoming, USA
2. Department of Ecosystem Science and Management, University of Wyoming, Laramie, Wyoming, USA
3. Department of Physics, Harvard University, Cambridge, Massachusetts, USA

Supplementary Figure 1


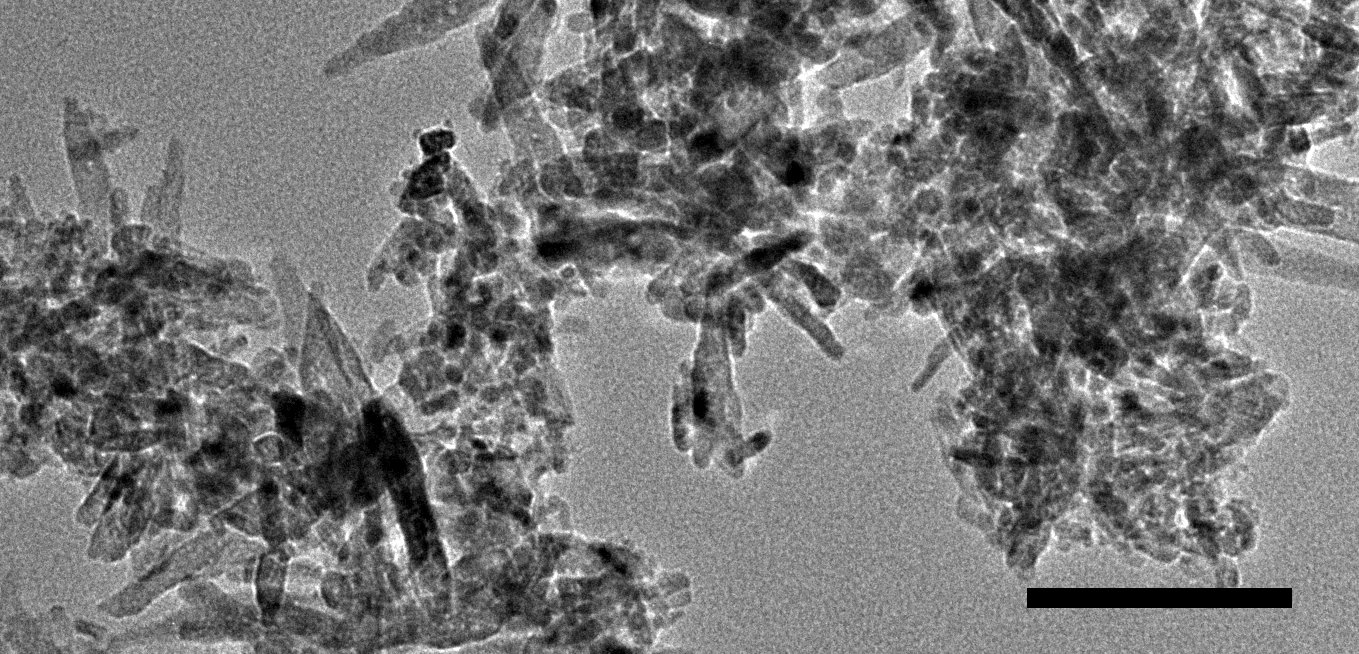


HRTEM image of CuO-NP. Black scale bar = 100nm (bottom right corner).

**Supplementary Figure 2**


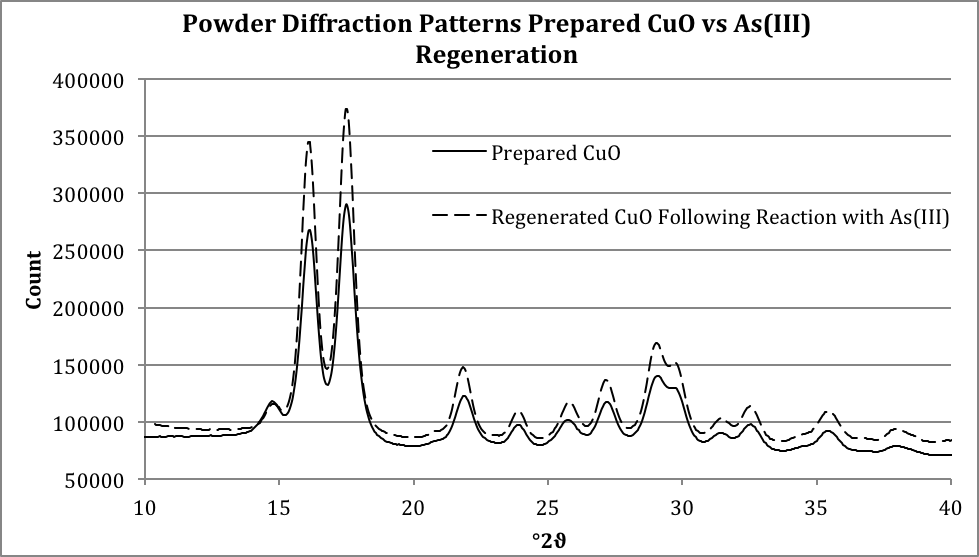


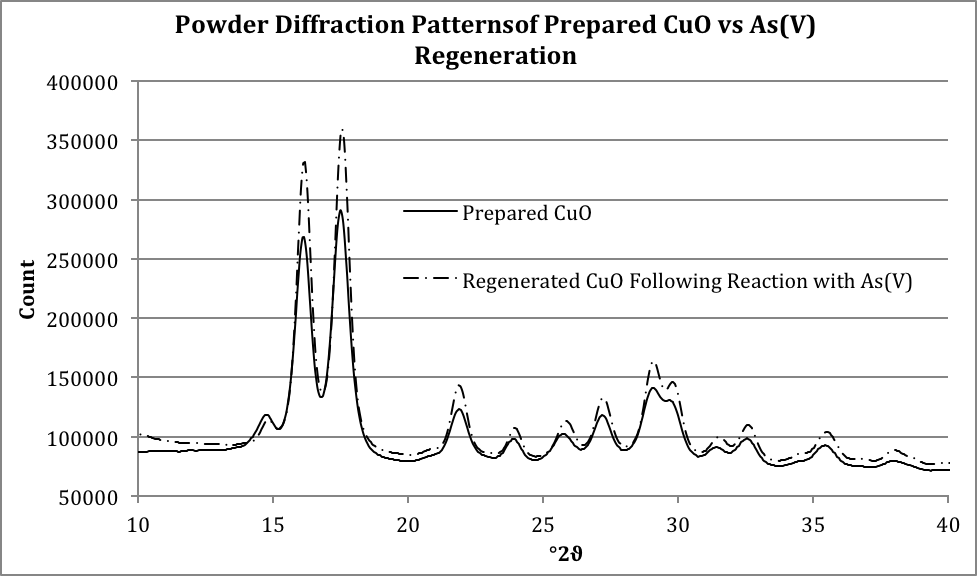


Powder X-ray diffraction patterns of CuO-NP as-prepared and following regenerations.

**Supplementary Figure 3**


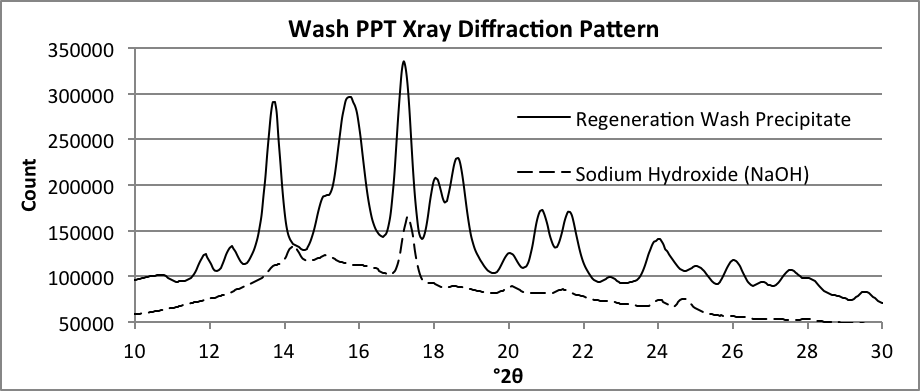

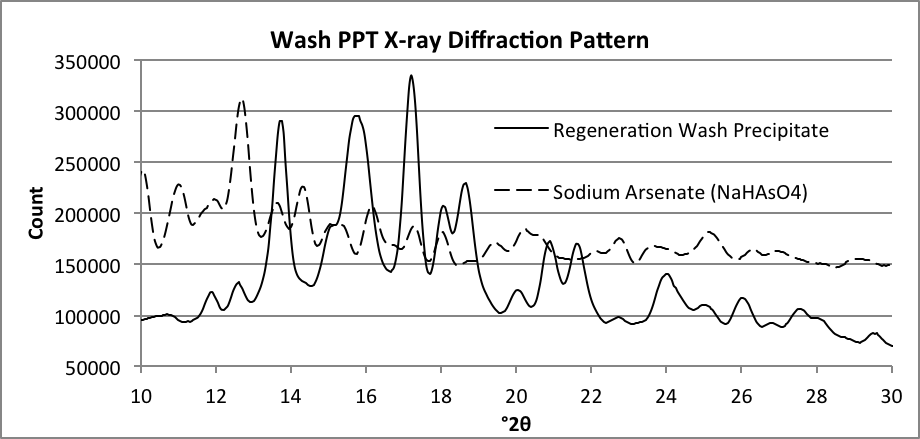

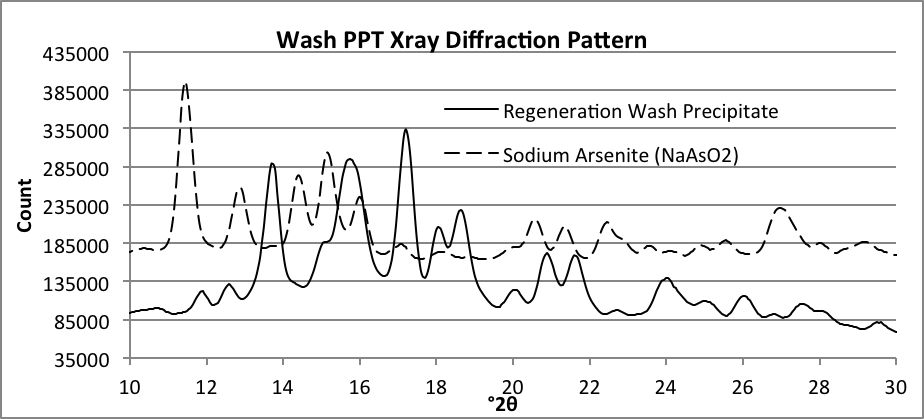
XRD patterns of regeneration wash fluid precipitate (PPT) compared to XRD patterns for sodium hydroxide, sodium arsenate, and sodium arsenite.

**Supplementary Figure 4**


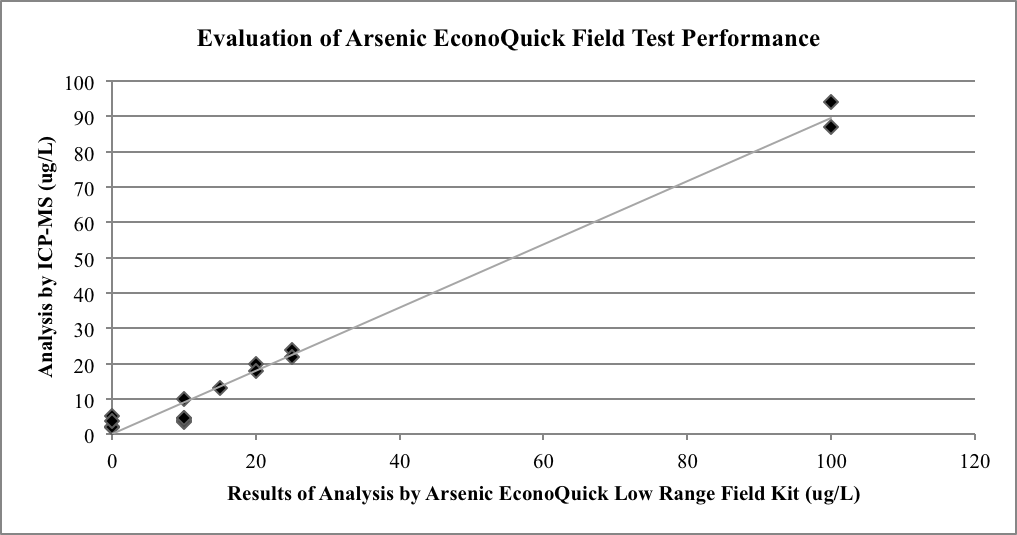


An evaluation of the performance of the Arsenic Econo-Quick Low Range field test kit as a real time monitor of arsenic concentrations used in conjunction with the point-of-use filter. The plot shows arsenic concentrations as analyzed by ICP-MS vs. on site analysis using the field kit for 20 select samples.

**Supplementary Table 1:** Mass balance of arsenic recovered in regeneration wash fluids.

|  | Description | pH | As (mg/L) | As (corrected after acidification) | Volume (mL) | Total As (mg) | Removed As (mg) | As Recovery (%) |
| --- | --- | --- | --- | --- | --- | --- | --- | --- |
| Primary | Prepared water Spiked to ~200 mg/L As | 10.05 | 180 | - | 50 | 9.0 | - | 85% |
|  | Post Treatment | 7.88 | 110 | - | 50 | 5.5 | 3.5 |  |
|  | Wash Fluids | 12.04 | 12 | 11.9 | 250 | 3.0 | - |  |
| Duplicate | Prepared water Spiked to ~200 mg/L As | 10.08 | 160 | - | 50 | 8.0 | - | 99% |
|  | Post Treatment | 7.93 | 90 | - | 50 | 4.5 | 3.5 |  |
|  | Wash Fluids | 12.08 | 14 | 13.9 | 250 | 3.5 | - |  |
| Triplicate | Prepared water Spiked to ~200 mg/L As | 10.08 | 170 | - | 50 | 8.5 | - | 106% |
|  | Post Treatment | 8.05 | 100 | - | 50 | 5.0 | 3.5 |  |
|  | Wash Fluids | 12.13 | 15 | 14.9 | 250 | 3.7 | - |  |
|  |  |  |  |  |  |  | Average Recovery | = 96.6% |

**Supplementary Table 2:** Chemical characterization of Torrington groundwater and Jackson groundwater prior to treatment with the CuO-NP. A = Lab spiked groundwater. B = Torrington groundwater. C = Jackson groundwater. Units in μg/L. Iron is <0.02 μg/L. Lead is <0.8 μg/L. Chromium is <11 μg/L.

| **Sample** | **pH** | **As** | **Ca** | **Cu** | **Mg** | **Mn** | **K** | **Se** | **Na** | **Si** | **Cl** | **N** | | **P** | | **SO4** |
| --- | --- | --- | --- | --- | --- | --- | --- | --- | --- | --- | --- | --- | --- | --- | --- | --- |
| **A** | 7.56 | 94 | 72000 | 12 | 15000 | 2 | 1000 | 1 | 4100 | 4.9 | 11000 | | 6200 | 500 | 19000 | |
| **B** | 7.22 | 13 | 76000 | 10 | 8400 | 2 | 9500 | 3.5 | 51000 | 26 | 16000 | | 3300 | 500 | 150000 | |
| **C** | 7.50 | 24 | 41000 | 26 | 5800 | 2 | 1000 | 1 | 27027 | 7.7 | 9100 | | 200 | 500 | 15000 | |

**Supplementary Table 3:** Chemical data of sand only control with point-of-use filter for (A) lab-based spiked groundwater, (B) Torrington, and (C) Jackson trials. Due to costs associated with analysis, only arsenic was analyzed for samples taken throughout the flow through process.

| **Sample Time** | **Volume Treated** | **pH** | **As** | **Si** | **Ca** | **Cr** | **Cu** | **Fe** | **Pb** | **Mg** | **Mn** | **K** | **Se** | **Na** | **Cl** | **N** | **P** | **SO4** |
| --- | --- | --- | --- | --- | --- | --- | --- | --- | --- | --- | --- | --- | --- | --- | --- | --- | --- | --- |
| **min** | **L** | **-** | **µg/L** | | | | | | | | | | | | **mg/L** | | | |
| **A**=Pre-Treatment | 0 | 7.31 | 95 | 4.7 | 74000 | 10 | 18 | 100 | 0.5 | 15000 | 2 | 1000 | 1 | 4200 | 11 | 5.8 | 0.5 | 19 |
| 0 | 0.0 | 6.75 | 16 | - | - | - | - | - | - | - | - | - | - | - | - | - | - | - |
| 5 | 0.8 | 7.29 | 61 | - | - | - | - | - | - | - | - | - | - | - | - | - | - | - |
| 10 | 1.7 | 7.33 | 75 | - | - | - | - | - | - | - | - | - | - | - | - | - | - | - |
| 15 | 2.5 | 7.34 | 75 | - | - | - | - | - | - | - | - | - | - | - | - | - | - | - |
| 20 | 3.3 | 7.36 | 82 | - | - | - | - | - | - | - | - | - | - | - | - | - | - | - |
| 30 | 5.0 | 7.35 | 79 | - | - | - | - | - | - | - | - | - | - | - | - | - | - | - |
| 60 | 10.0 | 7.33 | 89 | - | - | - | - | - | - | - | - | - | - | - | - | - | - | - |
| 90 | 15.0 | 7.32 | 88 | - | - | - | - | - | - | - | - | - | - | - | - | - | - | - |
| 120 | 20.0 | 7.37 | 94 | - | - | - | - | - | - | - | - | - | - | - | - | - | - | - |
| Composite | 20.0 | 7.45 | 87 | 4.7 | 73000 | 10 | 10 | 100 | 0.5 | 15000 | 2 | 1000 | 1 | 4100 | 10 | 5.7 | 0.5 | 19 |
| **B**=Pre-Treatment | 0.0 | 7.22 | 13 | 26 | 76000 | 10 | 10 | 100 | 0.7 | 8400 | 2 | 9500 | 3.5 | 51000 | 16 | 3.3 | 0.5 | 150 |
| 0 | 0.0 | 6.36 | 2 | - | - | - | - | - | - | - | - | - | - | - | - | - | - | - |
| 5 | 0.8 | 6.95 | 6.2 | - | - | - | - | - | - | - | - | - | - | - | - | - | - | - |
| 10 | 1.7 | 7.07 | 9.8 | - | - | - | - | - | - | - | - | - | - | - | - | - | - | - |
| 35 | 5.8 | 7.27 | 11 | - | - | - | - | - | - | - | - | - | - | - | - | - | - | - |
| 60 | 10.0 | 7.26 | 12 | - | - | - | - | - | - | - | - | - | - | - | - | - | - | - |
| 90 | 15.0 | 7.21 | 12 | - | - | - | - | - | - | - | - | - | - | - | - | - | - | - |
| 120 | 20.0 | 7.2 | 13 | - | - | - | - | - | - | - | - | - | - | - | - | - | - | - |
| Composite | 20.0 | 7.32 | 10 | 26 | 77000 | 10 | 10 | 100 | 0.7 | 8500 | 2 | 9200 | 2.6 | 50000 | 16 | 3.4 | 0.5 | 150 |
| **C**=Pre-Treatment | 0.0 | 7.5 | 24 | 7.7 | 41000 | 10 | 26 | 100 | 0.5 | 5800 | 2 | 1000 | 1 | 54000 | 9.1 | 0.2 | 0.5 | 15 |
| 0 | 0.0 | 6.98 | 2 | - | - | - | - | - | - | - | - | - | - | - | - | - | - | - |
| 5 | 0.8 | 7.53 | 13 | - | - | - | - | - | - | - | - | - | - | - | - | - | - | - |
| 10 | 1.7 | 7.47 | 19 | - | - | - | - | - | - | - | - | - | - | - | - | - | - | - |
| 35 | 5.8 | 7.61 | 24 | - | - | - | - | - | - | - | - | - | - | - | - | - | - | - |
| 60 | 10.0 | 7.64 | 24 | - | - | - | - | - | - | - | - | - | - | - | - | - | - | - |
| 90 | 15.0 | 7.65 | 24 | - | - | - | - | - | - | - | - | - | - | - | - | - | - | - |
| 120 | 20.0 | 7.68 | 25 | - | - | - | - | - | - | - | - | - | - | - | - | - | - | - |
| Composite | 20.0 | 7.61 | 22 | 7.6 | 43000 | 10 | 10 | 100 | 0.5 | 5800 | 2 | 1000 | 1 | 55000 | 9.1 | 0.2 | 0.5 | 16 |

**Supplementary Table 4:** Chemical data of point-of-use filter with prepared CuO-NP for (A) lab-based spiked groundwater, (B) Torrington, and (C) Jackson trials. Average of duplicate trials.

| **Sample Time** | **Volume Treated** | **pH** | **As** | **Si** | **Ca** | **Cr** | **Cu** | **Fe** | **Pb** | **Mg** | **Mn** | | | **K** | **Se** | | **Na** | **Cl** | **N** | **P** | **SO4** |
| --- | --- | --- | --- | --- | --- | --- | --- | --- | --- | --- | --- | --- | --- | --- | --- | --- | --- | --- | --- | --- | --- |
| **min** | **L** | **-** | **µg/L** | | | | | | | | | | | | | | | **mg/L** | | | |
| **A**=Pre-Treatment | 0.0 | 7.56 | 94 | 4.9 | 72000 | 10 | 12 | 100 | 0.5 | 15000 | | 2 | 1000 | | | 1 | 4100 | 11 | 6.2 | 0.5 | 19 |
| 0 | 0.1 | 6.69 | 2 | - | - | - | - | - | - | - | | - | - | | | - | - | - | - | - | - |
| 5 | 0.8 | 7.17 | 2 | - | - | - | - | - | - | - | | - | - | | | - | - | - | - | - | - |
| 10 | 1.7 | 7.33 | 2 | - | - | - | - | - | - | - | | - | - | | | - | - | - | - | - | - |
| 15 | 2.5 | 7.42 | 2 | - | - | - | - | - | - | - | | - | - | | | - | - | - | - | - | - |
| 20 | 3.3 | 7.53 | 2 | - | - | - | - | - | - | - | | - | - | | | - | - | - | - | - | - |
| 30 | 5.0 | 7.6 | 2.8 | - | - | - | - | - | - | - | | - | - | | | - | - | - | - | - | - |
| 45 | 7.5 | 7.6 | 5.9 | - | - | - | - | - | - | - | | - | - | | | - | - | - | - | - | - |
| 60 | 10.0 | 7.55 | 13 | - | - | - | - | - | - | - | | - | - | | | - | - | - | - | - | - |
| Composite | 10.0 | 7.61 | 4.4 | 4.7 | 72000 | 10 | 10 | 100 | 0.5 | 14000 | | 2 | 1000 | | | 1 | 4400 | 13 | 6.2 | 0.5 | 19 |
| **B**= Pre-Treatment | 0.0 | 7.22 | 13 | 26 | 76000 | 10 | 10 | 100 | 0.7 | 8400 | | 2 | 9500 | | | 3.5 | 51000 | 16 | 3.3 | 0.5 | 150 |
| 0 | 0.0 | 6.55 | 2 | - | - | - | - | - | - | - | | - | - | | | - | - | - | - | - | - |
| 5 | 0.8 | 7.02 | 2 | - | - | - | - | - | - | - | | - | - | | | - | - | - | - | - | - |
| 10 | 1.7 | 7.06 | 2 | - | - | - | - | - | - | - | | - | - | | | - | - | - | - | - | - |
| 20 | 3.3 | 7.11 | 2 | - | - | - | - | - | - | - | | - | - | | | - | - | - | - | - | - |
| 35 | 5.8 | 7.13 | 2.55 | - | - | - | - | - | - | - | | - | - | | | - | - | - | - | - | - |
| 60 | 10.0 | 7.21 | 2.75 | - | - | - | - | - | - | - | | - | - | | | - | - | - | - | - | - |
| Composite | 10.0 | 7.35 | 2 | 26 | 73000 | 10 | 10 | 100 | 0.6 | 8200 | | 2 | 9050 | | | 2.9 | 49500 | 18 | 3.3 | 0.5 | 145 |
| **C**=Pre-Treatment | 0.0 | 7.5 | 24 | 7.7 | 41000 | 10 | 26 | 100 | 0.5 | 5800 | | 2 | 1000 | | | 1 | 27027 | 9.1 | 0.2 | 0.5 | 15 |
| 0 | 0.0 | 6.58 | 2 | - | - | - | - | - | - | - | | - | - | | | - | - | - | - | - | - |
| 5 | 0.8 | 7.10 | 2 | - | - | - | - | - | - | - | | - | - | | | - | - | - | - | - | - |
| 10 | 1.7 | 7.24 | 2 | - | - | - | - | - | - | - | | - | - | | | - | - | - | - | - | - |
| 20 | 3.3 | 7.30 | 2.1 | - | - | - | - | - | - | - | | - | - | | | - | - | - | - | - | - |
| 35 | 5.8 | 7.36 | 2.2 | - | - | - | - | - | - | - | | - | - | | | - | - | - | - | - | - |
| 60 | 10.0 | 7.42 | 3.25 | - | - | - | - | - | - | - | | - | - | | | - | - | - | - | - | - |
| Composite | 10.0 | 7.4 | 2.05 | 7.5 | 40000 | 10 | 10 | 100 | 0.5 | 5850 | | 2 | 1000 | | | 1 | 55000 | 11 | 0.2 | 0.5 | 16 |

**Supplementary Table 5:** Chemical data of point-of-use filter for regenerated CuO-NP for the lab-based spiked groundwater, Torrington, and Jackson trials. Average of duplicate trials.

| **Sample Time** | **Volume Treated** | **pH** | **As** | **Si** | **Ca** | **Cr** | **Cu** | **Fe** | **Pb** | **Mg** | **Mn** | | | **K** | **Se** | | **Na** | **Cl** | **N** | **P** | **SO4** |
| --- | --- | --- | --- | --- | --- | --- | --- | --- | --- | --- | --- | --- | --- | --- | --- | --- | --- | --- | --- | --- | --- |
| **min** | **L** | **-** | **µg/L** | | | | | | | | | | | | | | | **mg/L** | | | |
| **A**=Pre-Treatment | 0.0 | 7.56 | 94 | 4.9 | 72000 | 10 | 12 | 100 | 0.5 | 15000 | | 2 | 1000 | | | 1 | 4100 | 11 | 6.2 | 0.5 | 19 |
| 0 | 10 | 7.72 | 7.6 | - | - | - | - | - | - | - | | - | - | | | - | - | - | - | - | - |
| 5 | 10.8 | 7.78 | 13 | - | - | - | - | - | - | - | | - | - | | | - | - | - | - | - | - |
| 10 | 11.7 | 7.69 | 14 | - | - | - | - | - | - | - | | - | - | | | - | - | - | - | - | - |
| 15 | 12.5 | 7.66 | 14 | - | - | - | - | - | - | - | | - | - | | | - | - | - | - | - | - |
| 20 | 13.3 | 7.66 | 16 | - | - | - | - | - | - | - | | - | - | | | - | - | - | - | - | - |
| 30 | 15.0 | 7.67 | 19 | - | - | - | - | - | - | - | | - | - | | | - | - | - | - | - | - |
| 45 | 17.5 | 7.63 | 27 | - | - | - | - | - | - | - | | - | - | | | - | - | - | - | - | - |
| 60 | 20.0 | 7.64 | 35 | - | - | - | - | - | - | - | | - | - | | | - | - | - | - | - | - |
| Composite | 20.0 | 7.89 | 20 | 4.6 | 75000 | 10 | 18 | 100 | 0.5 | 15000 | | 2 | 1000 | | | 1 | 4400 | 11 | 6 | 0.5 | 19 |
| **B**=Pre-Treatment | 10.0 | 7.22 | 13 | 26 | 76000 | 10 | 10 | 100 | 0.7 | 8400 | | 2 | 9500 | | | 3.5 | 51000 | 16 | 3.3 | 0.5 | 150 |
| 0 | 10.0 | 7.11 | 2 | - | - | - | - | - | - | - | | - | - | | | - | - | - | - | - | - |
| 5 | 10.8 | 7.38 | 2.25 | - | - | - | - | - | - | - | | - | - | | | - | - | - | - | - | - |
| 10 | 11.7 | 7.33 | 2.35 | - | - | - | - | - | - | - | | - | - | | | - | - | - | - | - | - |
| 20 | 13.3 | 7.35 | 3.45 | - | - | - | - | - | - | - | | - | - | | | - | - | - | - | - | - |
| 35 | 15.8 | 7.33 | 3.65 | - | - | - | - | - | - | - | | - | - | | | - | - | - | - | - | - |
| 60 | 20.0 | 7.37 | 5.25 | - | - | - | - | - | - | - | | - | - | | | - | - | - | - | - | - |
| Composite | 20.0 | 7.46 | 3.55 | 25 | 72500 | 10 | 15.5 | 100 | 0.58 | 8250 | | 2 | 9200 | | | 3.2 | 49000 | 16 | 3.2 | 0.5 | 145 |
| **C**=Pre-Treatment | 10.0 | 7.5 | 24 | 7.7 | 41000 | 10 | 26 | 100 | 0.5 | 5800 | | 2 | 1000 | | | 1 | 54000 | 9.1 | 0.2 | 0.5 | 15 |
| 0 | 10.0 | 7.19 | 2 | - | - | - | - | - | - | - | | - | - | | | - | - | - | - | - | - |
| 5 | 10.8 | 7.57 | 2.6 | - | - | - | - | - | - | - | | - | - | | | - | - | - | - | - | - |
| 10 | 11.7 | 7.63 | 2.15 | - | - | - | - | - | - | - | | - | - | | | - | - | - | - | - | - |
| 20 | 13.3 | 7.59 | 2.2 | - | - | - | - | - | - | - | | - | - | | | - | - | - | - | - | - |
| 35 | 15.8 | 7.52 | 3.1 | - | - | - | - | - | - | - | | - | - | | | - | - | - | - | - | - |
| 60 | 20.0 | 7.54 | 4.1 | - | - | - | - | - | - | - | | - | - | | | - | - | - | - | - | - |
| Composite | 20.0 | 7.64 | 2.95 | 6.8 | 37500 | 10 | 17.5 | 100 | 0.5 | 5600 | | 2 | 1000 | | | 1 | 50500 | 8.4 | 0.2 | 0.5 | 14.5 |

**Supplementary Table 6:** Chemical data of the wash fluids used to regenerate the CuO-NP with point-of-use filer using (A) lab-based spiked groundwater, (B) Torrington, and (C) Jackson trials. Average of duplicate trials. K, Fe, Cl, Cr, Mn, Pb, and Se concentrations were very low.

| **Sample** | Volume  (L) | Ca  (mg/L) | Mg  (mg/L) | Na  (mg/L) | Si  (mg/L) | Cl  (mg/L) | N  (mg/L) | P  (mg/L) | SO4  (mg/L) | Cu  (µg/L) | As  (µg/L) | %As Recovered |
| --- | --- | --- | --- | --- | --- | --- | --- | --- | --- | --- | --- | --- |
| **A** | 11.4 | 13.00 | 2.3 | 36.0 | 0.94 | 2 | 1.1 | 0.5 | 3.9 | 10.0 | 18 | 23 |
| **B** | 11.4 | 3.25 | 0.3 | 70.5 | 1.6 | 2.11 | 0.22 | 0.5 | 6.95 | 140.5 | 3.7 | 38 |
| **C** | 11.4 | 1.75 | 0.2 | 65.0 | 0.48 | 0.39 | 0.2 | 0.5 | 1 | 62.5 | 4.2 | 22 |

Supplementary Table 7: Detection limits of analytical instruments.

| **Analyte** | **Detection Limit**  **(mg/L)** | **Instrument** |
| --- | --- | --- |
| Sodium | 0.1 | Agilent ICP-MS-7500ce |
| Magnesium | 0.1 | Agilent ICP-MS-7500ce |
| Aluminum | 0.001 | Agilent ICP-MS-7500ce |
| Silicon | 0.1 | Agilent ICP-MS-7500ce |
| Potassium | 0.1 | Agilent ICP-MS-7500ce |
| Calcium | 0.1 | Agilent ICP-MS-7500ce |
| Chromium | 0.001 | Agilent ICP-MS-7500ce |
| Manganese | 0.001 | Agilent ICP-MS-7500ce |
| Iron | 0.02 | Agilent ICP-MS-7500ce |
| Copper | 0.001 | Agilent ICP-MS-7500ce |
| Zinc | 0.001 | Agilent ICP-MS-7500ce |
| Arsenic | 0.001 | Agilent ICP-MS-7500ce |
| Selenium | 0.001 | Agilent ICP-MS-7500ce |
| Lead | 0.001 | Agilent ICP-MS-7500ce |
| Cadmium | 0.001 | Agilent ICP-MS-7500ce |
| Phosphate | 0.1 | Dionex DX-500 |
| Sulfate | 0.5 | Dionex DX-500 |
| Nitrate | 0.05 | Dionex DX-500 |
| Chloride | 1 | Dionex DX-500 |
| Fluoride | 0.1 | Dionex DX-500 |
